# Facility-Wide Testing for SARS-CoV-2 in Nursing Homes — Seven U.S. Jurisdictions, March–June 2020

**DOI:** 10.15585/mmwr.mm6932e5

**Published:** 2020-08-11

**Authors:** Kelly M. Hatfield, Sujan C. Reddy, Kaitlin Forsberg, Lauren Korhonen, Kelley Garner, Trent Gulley, Allison James, Naveen Patil, Carla Bezold, Najibah Rehman, Marla Sievers, Benjamin Schram, Tracy K. Miller, Molly Howell, Claire Youngblood, Hannah Ruegner, Rachel Radcliffe, Allyn Nakashima, Michael Torre, Kayla Donohue, Paul Meddaugh, Mallory Staskus, Brandon Attell, Caitlin Biedron, Peter Boersma, Lauren Epstein, Denise Hughes, Meghan Lyman, Leigh E. Preston, Guillermo V. Sanchez, Sukarma Tanwar, Nicola D. Thompson, Snigdha Vallabhaneni, Amber Vasquez, John A. Jernigan

**Affiliations:** ^1^CDC COVID-19 Response Team; ^2^Arkansas Department of Health; ^3^Detroit Health Department, Detroit, Michigan; ^4^New Mexico Department of Health; ^5^North Dakota Department of Health; ^6^South Carolina Department of Health and Environmental Control; ^7^Utah Department of Health; ^8^Vermont Department of Health.

Undetected infection with SARS-CoV-2, the virus that causes coronavirus disease 2019 (COVID-19) contributes to transmission in nursing homes, settings where large outbreaks with high resident mortality have occurred (*1*,*2*). Facility-wide testing of residents and health care personnel (HCP) can identify asymptomatic and presymptomatic infections and facilitate infection prevention and control interventions (*3*–*5*). Seven state or local health departments conducted initial facility-wide testing of residents and staff members in 288 nursing homes during March 24–June 14, 2020. Two of the seven health departments conducted testing in 195 nursing homes as part of facility-wide testing all nursing homes in their state, which were in low-incidence areas (i.e., the median preceding 14-day cumulative incidence in the surrounding county for each jurisdiction was 19 and 38 cases per 100,000 persons); 125 of the 195 nursing homes had not reported any COVID-19 cases before the testing. Ninety-five of 22,977 (0.4%) persons tested in 29 (23%) of these 125 facilities had positive SARS-CoV-2 test results. The other five health departments targeted facility-wide testing to 93 nursing homes, where 13,443 persons were tested, and 1,619 (12%) had positive SARS-CoV-2 test results. In regression analyses among 88 of these nursing homes with a documented case before facility-wide testing occurred, each additional day between identification of the first case and completion of facility-wide testing was associated with identification of 1.3 additional cases. Among 62 facilities that could differentiate results by resident and HCP status, an estimated 1.3 HCP cases were identified for every three resident cases. Performing facility-wide testing immediately after identification of a case commonly identifies additional unrecognized cases and, therefore, might maximize the benefits of infection prevention and control interventions. In contrast, facility-wide testing in low-incidence areas without a case has a lower proportion of test positivity; strategies are needed to further optimize testing in these settings.

CDC compiled data from seven state or local health departments that conducted facility-wide testing in nursing homes. Testing of specimens (i.e., from the nasopharynx or anterior nares) for SARS-CoV-2 was performed using reverse transcription–polymerase chain reaction (RT-PCR) testing; one health department also used point-of-care testing with Abbott ID Now (Abbott Diagnostics, Inc.). Two health departments conducted initial facility-wide testing in all nursing homes in the state (i.e., statewide testing strategy). Five health departments targeted initial facility-wide testing to facilities with a newly reported case in a resident or HCP (i.e., targeted testing strategy). Five nursing homes were included because of high COVID-19 incidence in the surrounding county or a neighboring nursing home outbreak. For each testing event, all orally consenting residents and HCPs (*6*) at a facility were tested. Results are reported at the individual level, thus if a resident or HCP had more than one positive test result, they were only included once.

Because testing strategies varied by health department, data were aggregated according to testing strategy. Results were stratified by resident and HCP status when possible. County-level cumulative COVID-19 incidence for the 14 days preceding testing was calculated for each facility, using information from USAFacts.* For facilities using the targeted testing strategy, a linear generalized estimating equation (GEE) was used to estimate the association between the number of days from identification of the first COVID-19 case in the nursing home until completion of the facility-wide testing and the cumulative number of persons with positive SARS-CoV-2 test results, adjusting for the number of persons tested and the surrounding county incidence. For a subset of 62 facilities using the targeted strategy with data on resident and HCP status, a GEE model was used to describe the relationship between the cumulative number of residents and HCP with positive SARS-CoV-2 test results at completion of the initial testing, adjusting for the number of residents and HCP tested and the county incidence. Models were fitted using GEE with an exchangeable correlation structure that accounted for clustering within jurisdictions (*7*). In the statewide testing strategy group, associations were assessed between the COVID-19 incidence in the surrounding county and the odds of identifying any cases at each facility testing event, adjusted for the number of persons tested in all facilities that did not have previous cases. Logistic GEE models with an exchangeable correlation structure accounting for clustering by jurisdiction (*7*) were fitted. The role of facility size was not assessed, but in the multivariable models, adjustment was made for the number of persons who received testing as a proxy for facility size. All analyses were conducted using SAS (version 9.4; SAS Institute); statistical significance was assessed using p<0.05. This investigation was deemed not human subjects research under Department of Health and Human Services, Title 45 Code of Federal Regulations 46, Protection of Human Subjects.

Overall, seven health departments provided data from 288 nursing homes that conducted initial facility-wide testing during March 24–June 14 (Table 1). Health departments reported turnaround times ranging from 1 to 7 days from testing until receipt of results.

**TABLE 1:** Characteristics of nursing homes that completed facility-wide testing for SARS-CoV-2, by testing strategy and health department (N = 288) — seven state and local health department jurisdictions, United States, March 24–June 14, 2020

Characteristic	Targeted testing strategy*					Statewide testing strategy*	
	Arkansas	Detroit, Michigan^†^	New Mexico	Utah	Vermont	North Dakota	South Carolina
No. of nursing homes	29	26	16	16	6	50	145^§^
No. of counties represented	19	1	8	4	4	33	41
No. (%) of known COVID-19 cases before facility-wide testing	29 (100)	26 (100)	11^¶^ (69.0)	16 (100)	6 (100)	11 (22.0)	59 (41.0)
No. of patients tested	5,039	2,550	3,139	2,227	488	8,728	28,737
No. (%) of cases after facility-wide testing	184 (3.7)	1,048 (41.1)	166 (5.3)	149 (6.7)	72 (14.8)	93 (1.1)	333 (1.1)
No. of persons tested per facility, median (range)	159 (83–349)	94.5 (44–161)	194 (71–322)	92 (15–436)	74 (22–150)	126 (29–504)	186 (20–792)
No. of cases per facility before facility-wide testing, median (range)	2 (1–15)**	12.5 (2–32)	1 (0–21)	2 (1–10)	1 (1–30)	Unknown	Unknown
No. cases per facility at completion of facility-wide testing, median (range)	2 (1–52)	35 (14–99)	2.5 (0–51)	6.5 (1–33)	2 (1–51)	0 (0–19)	0 (0–45)
Dates of 2020 facility-wide testing completion, range (span, days)	Mar 24– Apr 26 (33)	Apr 16– Apr 25 (9)	Apr 2– May 5 (33)	Mar 31– Jun 14 (75)	Mar 30– Apr 22 (23)	Apr 10– Jun 4 (24)	May 4– Jun 5 (32)
Days from first case to testing per facility, median (range)	5 (1–17)	32 (20–41)	8 (1–17)	4 (1–12)	6 (2–18)	5 (4–32)^††^	30 (1–66)
Incidence^§§^ per facility in surrounding county, median (IQR)	28 (13–52)	282 (280–322)	43 (32–117)	91 (57–100)	72 (64–105)	19 (0–38)	38 (21–72)

**Abbreviations:** COVID-19 = coronavirus disease 2019; IQR: interquartile range.

* Targeted testing strategy represents health departments that performed facility-wide testing of residents and health care personnel in response to a known or suspected case. Statewide testing strategy represents health departments that conducted facility-wide testing statewide.

^†^ Health care personnel data were not available from the Detroit Health Department for this analysis. The Detroit Health Department used the Abbot ID Now (Abbott Diagnostics, Inc.) for some tests reported; all others used reverse transcription–polymerase chain reaction testing.

^§^ Persons in 194 nursing homes received testing as part of statewide testing efforts; 145 nursing homes included in this analysis had reported complete aggregate data to their respective health department as of July 14, 2020.

^¶^ Eleven nursing homes conducted testing in response to a known case; five nursing homes performed testing in response to high county incidence or nearby outbreaks (no previously identified cases of coronavirus disease 2019 [COVID-19] in that nursing home). ** Number of cases before the facility-wide testing was unknown for four facilities.

^††^ Unknown for eight of 11 nursing homes with known cases of COVID-19 before facility-wide testing.

^§§^ The cumulative number of new cases in the county per 100,000 population in the 14 days before the facility-wide testing. Data from USAfacts (https://usafacts.org/) was used to calculate county incidence.

Five health departments using the targeted testing strategy (Arkansas; Detroit, Michigan; New Mexico; Utah; and Vermont) tested 93 nursing homes, and in 79% of those, new COVID-19 cases were detected (median = 6 new cases, interquartile range = 1–21). In these 93 nursing homes, 13,443 persons were tested, and 1,619 (12%) had positive SARS-CoV-2 test results. Among the 93 nursing homes, 88 (95%) had a documented COVID-19 case before testing; the number of days between identification of the first case and the completion of facility-wide testing ranged from 1 to 41 days (median = 7 days). Population average estimates from regression analyses suggested that each additional day from case identification to facility-wide testing was associated with identification of 1.3 additional cases (Figure). Among 62 facilities for which resident and HCP results could be differentiated, a linear association was found between the number of residents and HCP who had positive SARS-CoV-2 testing results (p<0.001): an estimated 1.3 cases among HCP were identified for every three resident cases. In 45 (73%) of these facilities with at least one resident with test results positive for SARS-CoV-2, an average of 5.2% HCP who were tested had positive test results (range = 0%–26%).

**FIGURE:**
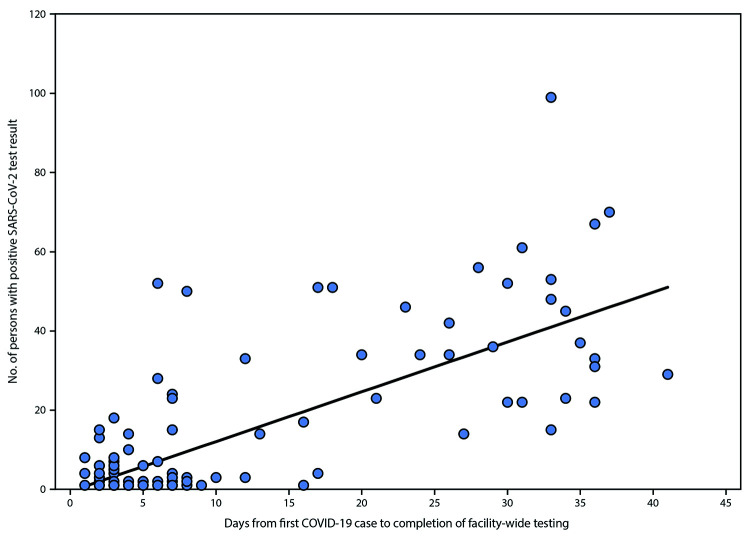
Association between total number of persons with positive SARS-CoV-2 test results after facility-wide testing and number of days from first case identification until completion of facility-wide testing* — five state and local health department jurisdictions,^†^ United States, March–June 2020 **Abbreviation:** COVID-19 = coronavirus disease 2019. * The parameter estimate, based on generalized estimating equations modeling the relationship of days from first case of COVID-19 in a nursing home to completion of facility-wide testing, was 1.3 (95% CI = 1.0–1.5) and was adjusted for the surrounding county incidence and the total number of persons tested during facility-wide testing. This parameter was separately estimated excluding facilities in Detroit, which used the Abbot ID Now platform and produced similar results (parameter estimate = 1.3; 95% CI = 0.6–2.0). All other sites used reverse transcription–polymerase chain reaction testing. ^†^ The five jurisdictions (Arkansas; Detroit, Michigan; New Mexico; Utah, and Vermont) used a targeted testing strategy.

The two health departments using a statewide testing strategy (North Dakota and South Carolina) conducted facility-wide testing in 195 nursing homes in low-incidence areas (i.e., the median preceding 14-day cumulative incidence in the surrounding county for each jurisdiction was 19 and 38 cases per 100,000 persons). Seventy (36%) of the 195 nursing homes had reported one or more residents or HCP with positive SARS-CoV-2 test results before the testing event, whereas 125 (64%) had not reported cases. Among 22,977 persons tested at the 125 nursing homes that had not reported cases, 95 (0.4%) had positive test results; 29 (23%) facilities each identified one to 25 cases, including 23 (18%) with one to three cases, and six (5%) with four or more cases. Multivariable models found no association between the cumulative county incidence and the odds of identifying a case among these 125 nursing homes (p = 0.67). Within the 70 nursing homes that reported cases in residents or HCP before the facility-wide testing, 14,488 persons were tested, and 331 (2%) had a positive result. For 62 facilities with available data, the number of days between identification of the first case and the facility-wide testing ranged from 1 to 66 days (median = 29.5 days). However, the cumulative number of cases was not available. Among the 70 facilities, 41 (59%) identified one to 45 cases, including 21 (30%) that identified one to three cases and 20 (29%) that identified four or more cases.

With both testing strategies, the mean number of cases identified in nursing homes was higher among those with at least one resident case identified before the facility-wide testing (25.7 among those using a targeted testing strategy, 7.3 among those using a statewide testing strategy), compared with those that had previously identified only HCP cases (3.5 and 0.3, respectively) or had no known cases before the testing (0.8 and 0.4, respectively) (p<0.001) (Table 2).

**TABLE 2:** Number of COVID-19 cases identified in nursing homes that conducted facility-wide SARS-CoV-2 testing as part of a statewide strategy targeting all nursing homes (statewide strategy) and those that conducted facility-wide testing only after identification of a known or suspected case (targeted strategy), by resident or health care provider cases identified before facility-wide testing — seven state and local health department jurisdictions, United States, March–June, 2020

Types of cases known before testing	Statewide testing strategy*			Targeted testing strategy^†^		
	No. of nursing homes^§^	No. of persons with positive test results^¶^		No. of nursing homes**	No. of persons with positive test results^¶^	
		Mean (SD)	Range		Mean (SD)	Range
One or more residents	35	7.3 (11.2)	0–45	59	25.7 (21.9)	1–99
Health care personnel only	22	0.3 (0.6)	0–2	22	3.5 (3.2)	1–13
No cases known	125	0.8 (2.7)	0–25	5	0.4 (0.9)	0–2

**Abbreviations:** COVID-19 = coronavirus disease 2019; SD = standard deviation.

* Conducted in two health department jurisdictions (North Dakota and South Carolina).

^†^ Conducted in five health department jurisdictions (Arkansas; Detroit, Michigan; New Mexico; Utah; and Vermont).

^§^ Thirteen nursing homes from the statewide strategy are excluded because the quantification of health care personnel cases and resident cases before the facility-wide testing was not possible.

^¶^ At completion of facility-wide testing.

** Seven nursing homes from the targeted strategy are excluded because the quantification of health care personnel cases and resident cases before the facility-wide testing was not possible.

## Discussion

Facility-wide testing of residents and HCP in nursing homes can provide important insights into the epidemiology of SARS-CoV-2 transmission and permit early identification of cases to guide infection prevention and control interventions. Conducting facility-wide testing as soon as possible after identifying a case of COVID-19 offers advantages over other approaches. First, previously undetected cases can be identified; these data indicate that 79% of testing events performed in response to a known case identified unrecognized cases. Second, testing as soon as possible after identifying an initial case was associated with identification of fewer cases and might improve the feasibility and effectiveness of cohorting (i.e., designating a location and HCP exclusively for care of residents with COVID-19) and other isolation strategies aimed at interrupting transmission (*8*). For these reasons, testing of all residents and HCP in a nursing home with efficient turnaround time is recommended as soon as possible after identifying a new COVID-19 case (*6*,*9*).

An association was found between infections in residents and infections in HCP, and the prevalence of infections among HCP was often higher than expected given results of community serosurveys in low-incidence settings, raising the possibility that infections in HCP might be occurring in the workplace (*10*). Transmission likely occurred between residents and HCP and among HCP, highlighting the importance of testing both residents and HCP to detect virus transmission and the need for more effective interventions to prevent transmission among HCP working in nursing homes.

Testing guidance for nursing homes has suggested baseline testing of all residents and serial testing of HCP as part of the “reopening process” (e.g., the relaxing of restrictions) (*6*,*8*). In low-incidence areas a large number of tests was needed to identify a few cases (0.4% persons with positive test results in places that had never had a COVID-19 case). In facilities without known COVID-19 cases, strategies to improve testing efficiency might focus on populations at highest risk for acquisition (e.g., HCP living in high-incidence areas or residents who might have been recently exposed during hospitalization or dialysis treatments). Other methods to improve efficiency might include point-of-care testing with rapid turnaround time, sample pooling, self-collection of samples (e.g., saliva or anterior nares swabs), or wastewater surveillance.

The findings in this report are subject to at least four limitations. First, symptoms at the time of testing were not systematically collected; thus, determining what proportion of cases might have been identified using symptom screening methods is not possible. Second, it was not possible to describe variations in infection prevention and control, other interventions that might affect COVID-19 spread, or follow-up over time. The full effectiveness of facility-wide testing (and total number of cases identified) might only be known through follow-up testing. Cases might be missed if the patient was no longer shedding virus, still incubating disease, or if less sensitive tests, such as point-of-care tests, are used. In this report, one health department used the less sensitive Abbott ID Now for some testing; however, findings were consistent when excluding that jurisdiction’s data.^†^ Third, the estimates of the relationship between cases identified and delays in conducting testing might only be relevant for the period examined (i.e., 1–41 days); this relationship might not be valid for longer delays as the number of persons susceptible to infection decreases. Finally, health departments contributing statewide testing data had a relatively low community incidence at time of testing; findings from jurisdictions with a higher community incidence might differ.

These observations from facility-wide testing in nursing homes in seven U.S. health jurisdictions can inform use of test-based prevention strategies in these settings. Facility-wide testing after identification of an index case might maximize the benefits of infection prevention and control interventions by enabling early identification of unrecognized cases, cohorting and isolation of resident cases, and exclusion of infected HCP from the workplace through nonpunitive sick-leave policies. Facility-wide testing in low-incidence areas without a case has a lower proportion of test positivity; strategies are needed to optimize testing in these nursing homes. State and local health departments need to take steps to ensure that nursing homes have the resources necessary to rapidly perform facility-wide testing among residents and HCP after identification of a case.

**Summary**

**What is already known about this topic?**

Facility-wide testing of health care personnel and nursing home residents for SARS-CoV-2 can inform strategies to prevent transmission.

**What is added by this report?**

In two health department jurisdictions, testing in facilities without a previous COVID-19 case identified a prevalence of 0.4%. Five health department jurisdictions that targeted facility-wide testing after identification of a case found a prevalence of 12%; for each additional day before completion of initial facility-wide testing, an estimated 1.3 additional cases were identified.

**What are the implications for public health practice?**

Performing facility-wide testing rapidly following identification of a case in a nursing home might facilitate control of transmission among residents and health care personnel. Strategies are needed to optimize facility-wide testing in nursing homes without a reported case.

## Footnotes

* https://usafacts.org/visualizations/coronavirus-covid-19-spread-map/.

† When excluding nursing homes from Detroit, which used Abbot ID Now for testing, the findings that for each additional day before completion of an initial facility-wide testing, 1.3 additional cases were identified and that the mean number of persons who had positive test results at the completion of facility-wide testing was highest among facilities with one or more resident cases before the testing event were consistent.
